# Fatigue Risk Management: A Maritime Framework

**DOI:** 10.3390/ijerph13020175

**Published:** 2016-01-29

**Authors:** Michelle Rita Grech

**Affiliations:** Australian Maritime Safety Authority, 82 Northbourne Avenue, Braddon ACT 2612, Australia; michelle.grech@amsa.gov.au; Tel.: +61-2-6279-5045

**Keywords:** maritime, fatigue, work hours, rest hours, shipping, fatigue risk management, FRMS, safety management, safety assurance, maritime fatigue

## Abstract

It is evident that despite efforts directed at mitigating the risk of fatigue through the adoption of hours of work and rest regulations and development of codes and guidelines, fatigue still remains a concern in shipping. Lack of fatigue management has been identified as a contributory factor in a number of recent accidents. This is further substantiated through research reports with shortfalls highlighted in current fatigue management approaches. These approaches mainly focus on prescriptive hours of work and rest and include an individualistic approach to managing fatigue. The expectation is that seafarers are responsible to manage and tolerate fatigue as part of their working life at sea. This attitude is an accepted part of a seafarer’s role. Poor compliance is one manifest of this problem with shipboard demands making it hard for seafarers to follow hours of work and rest regulations, forcing them into this “poor compliance” trap. This makes current fatigue management approaches ineffective. This paper proposes a risk based approach and way forward for the implementation of a fatigue risk management framework for shipping, aiming to support the hours of work and rest requirements. This forms part of the work currently underway to review and update the International Maritime Organization, Guidelines on Fatigue.

## 1. Introduction

### 1.1. Background

Shipping, like other transport industries (rail, aviation, commercial road transport) works a twenty-four hour continuous operation. Prescriptive hours of work and rest limits set out in the International Maritime Organization (IMO) and the International Labour Organization (ILO) Conventions are considered to be the primary fatigue risk management requirements, setting minimum standards of compliance in international shipping. Current regulations allow a maximum of 14 h of work in a 24-h period with a maximum of 72 h of work in a 7 day period [1,2]. IMO regulations also set minimum rest periods which should not be less than 10 h in any 24-h period and 77 h in any 7 day period. The rest period can be divided into no more than two periods, one of which shall be at least 6 h in length [2]. Recent studies identified that compliance in meeting these regulatory requirements in the maritime industry is generally poor, with differences observed in the actual and recorded hours of work and rest [3,4,5].

As shipping functions in a highly competitive environment, ship owners and/or operators are compelled to seek economic efficiencies, sometimes through reduction in the number of shipboard crew [6]. As seafarers endeavor to cope with increased demands due to reduced crew size they are consequently exposed to more demanding work conditions [7]. The operational aspects associated with shipping become more problematic compared with other industries for reasons such as: trading patterns, length of sea passage, port-rotation, and the short length of time a ship remains in port, all contributing to compressing seafarers’ recovery periods [3,4]. The increasingly intensive nature of shipping operations means that seafarers have to work long and irregular hours across long periods of time and are frequently subjected to; restricted and interrupted sleep, rapidly rotating shifts, high workload, poor eating habits, poor sleeping conditions, social isolation and no clear separation between work and recreation [3,4,7,8,9,10].

Current shipboard demands and working arrangements at sea are now being viewed as problematic creating a situation that do not allow seafarers adequate opportunity for rest and sleep [3,11,12]. Most commonly, seafarers are willing to work whilst highly fatigued because it is seen as “professional” to do so. The widely held belief that fatigue “comes with the job”, whilst not particular to the maritime industry is certainly pervasive within it, making it difficult for seafarers to recognise fatigue as a problem and to take appropriate action. This “forces” crew into the poor compliance trap as they feel responsible to manage their own shipboard fatigue [12,13], making current fatigue management approaches ineffective. Hence, the employment of a multi-layered defensive strategy to manage fatigue-related risks at sea is critical. This allows fatigue to be managed in the same way as any other shipboard hazard, using a risk based approach with proper company support. This paper proposes a way forward for the implementation of a fatigue risk management framework for maritime using the system defences of barriers and safeguards referred to as the “defences in depth” model. This provides for multiple layers of defences with a comprehensive, proactive approach to managing the risks of fatigue at sea.

Bearing in mind that ninety percent of world goods are transported by sea, shipping has and will continue to have significant economic, environmental and social value. With around 1.2 million seafarers working in international shipping, focusing on a risk based approach to fatigue management at sea is now seen as critically vital not only for their safety but also their long term health and general well-being [13,14,15].

### 1.2. Fatigue and Shipping Accidents

In 2011 the Australian Transport Safety Bureau published an investigation report into the grounding of the Bulk carrier *Shen Neng 1* in which the “lack of fatigue management processes” was cited as a contributory factor. The narrative below is based on information contained in this report [16].

On the 3 April 2010, at 1705 the bulk carrier *Shen Neng 1* grounded on Douglas Shoal while travelling outbound, about 50 miles from the port of Gladstone, Australia [16]. Although no injuries were sustained, the ship’s hull was badly damaged and breached, resulting in pollution. The accident occurred in a particularly sensitive and environmental protected area which in turn brought with it high media attention. A missed course alternation by the chief mate, on bridge duty at the time, resulted in the grounding. The chief mate was a competent seafarer with more than 20 years-experience on seagoing vessels. The question was hence raised—“*How could an experienced and competent senior officer miss such a critical task in the course of his duty?*”

The bridge crew on the *Shen Neng 1* operated a three watch duty cycle of 4-h on, 8-h off. The chief mate was assigned duty periods between the hours of 1600 to 2000 and 0400 to 0800. He had recently joined the vessel and it was his first time entering the port of Gladstone. To ensure smooth loading, he spent an extra three hours on duty following his evening watch (1600–2000) to check cargo and stability calculations. He did this for three days before the ship was due to enter port. This restricted his sleep opportunity to less than five hours per night (between 2300–0400). On the 2 April, as part of his port entry responsibilities the chief mate started work at 0300. From when the ship came alongside onwards, he remained on duty to supervise loading and de-ballasting operations, and by midnight of the 2 April he had been awake for around 22 h. It was 0100 on the 3 April, by the time the chief mate went to his cabin to sleep. He was woken up two hours later, at 0300, to resume duties and prepare the ship for its outbound transit. By the time he got back to his cabin for a rest it was 1220 (around 9.5 h later). He managed to fall asleep at 1500, but was woken up by his alarm clock 30 min later (1530), set in preparation for his next assigned watch schedule (1600–2000). He was on the bridge by 1550 for the watch hand-over from the second mate. Although he did not have adequate sleep (*i.e.*, only around 2.5 h sleep in the previous 37.5 h), the chief mate did not say anything about feeling tired to the second mate or the master. Prior to the watch hand-over, the second mate fixed the position of the *Shen Neng 1* on the chart and pointed out the next way point (next ship’s course alteration) to the chief mate.

The weather was calm with good visibility. There was no traffic in the area and little communication was had between the chief mate and the lookout (the only other person on the bridge at the time). The chief mate planned to take a fix of the ship’s position at 1630, however the chief engineer came to the bridge exactly at this time to request the ship’s speed and henceforth the chief mate decided to defer this task for later (around 1700). At 1642 however, the ship had already passed the next course alteration waypoint. At 1700, the chief mate recorded the position on the chart. By the time he realized, from the chart position, that the ship was entering the boundary area into a shoal, it was too late. At 1705 the vessel grounded.

At the time of the grounding the chief mate had slept for 2.5 h in the preceding 38.5 h. From when he took over the watch at 1600 until the grounding he displayed impaired reasoning and poor decision making, typical signs and symptoms of fatigue [17,18].

### 1.3. Fatigue Management and the Seafarer

What happened on this ship, in terms of the chief mate’s working hours, sleep opportunity and consequential behaviors is not unusual in shipping [5,13]. In this case the situation resulted in a succession of errors made by the chief mate as a result of fatigue, which eventually led to the grounding. In many other accidents in which fatigue was identified as a safety issue, crew fell asleep while on duty at critical times of the voyage with negative consequential outcomes [19,20,21]. This aspect is supported by research findings in which seafarers reported and were recorded falling asleep whilst on duty [11,12].These accidents demonstrate the high price paid in human loss, environmental pollution and material damage as a result of shipboard-work related fatigue [10].

Of biggest concern identified through the *Shen Neng 1* accident is the chief mate and the other crew’s attitude to inadequate sleep and long and excessive working hours. Most probably, as is common practice on ships, the chief mate thought he was doing the right thing by spending extra hours after his evening anchor watch on this task. It is not unusual for chief mates on most ships to work the extra hours to ensure that loading/unloading operations are completed without undue delays. Furthermore, chief mates and possibly other seafarers often need to be present for cargo operations and inspections (e.g., by port and state authorities, classification societies) which in most cases are done during the night to maximize charter times. This makes working while the ship is in port highly demanding [4]. This restricted the opportunity for the chief mate on the *Shen Neng 1* to obtain adequate sleep. The chief mate however continued and was permitted to relieve the second mate, even though he was not fit to conduct the 1600 to 2000 watch safely. This reflects a common culture in shipping in which seafarers continue working irrespective of prior hours of wakefulness and/or sleep quantity and quality [3,13]. In some cases recording hours of work which do not reflect actual hours worked [4,14]. What the *Shen Neng 1* grounding and other maritime accidents reveal is that seafarers are usually left to their own devices to manage their fatigue levels. In most cases, they would simply, and as part of “their job”, record their hours of work in a way which would not reflect badly on themselves, the master or the company [13].

The Authority investigating the *Shen Neng 1* primarily recommended the requirement for the ship’s management company to have a fatigue management system in place. It is not known whether these recommendations were implemented, however, there is now general consensus that a renewed effort, through the International Maritime Organization (IMO) needs to be adopted to ensure that seafarer fatigue is managed effectively [13,14,22].

## 2. Development of Fatigue Risk Management for Shipping

### 2.1. Current Approach to Fatigue Management at Sea

A regulatory prescriptive approach to managing fatigue is adopted in international shipping. Maximum work hours and/or minimum rest periods are specified [1,2]. Under these regulations, written individual records are required to be kept for auditing and inspection purposes. However, as was the case in the *Shen Neng 1*, these records are often under reported as seafarers work longer hours to meet the demands of operational commitments [3,13]. As suggested in the literature, prescribed hours of work and rest requirements represent one layer of defence and alone, are not enough to mitigate and control the risks of fatigue [23,24]. While complying with these requirements is a necessary part of managing fatigue risks, these regulations do not employ any systematic or scientific basis for assessing risk and applying safety thresholds. Hence, supplementary defensive layers are necessary to manage the fatigue risks effectively. Research consistently indicates that seafarers rated a lack of sleep and inconsistent sleep times to be the leading contributors to fatigue, suggesting that the opportunity for adequate sleep is not provided [4,10,18].

**Figure 1:**
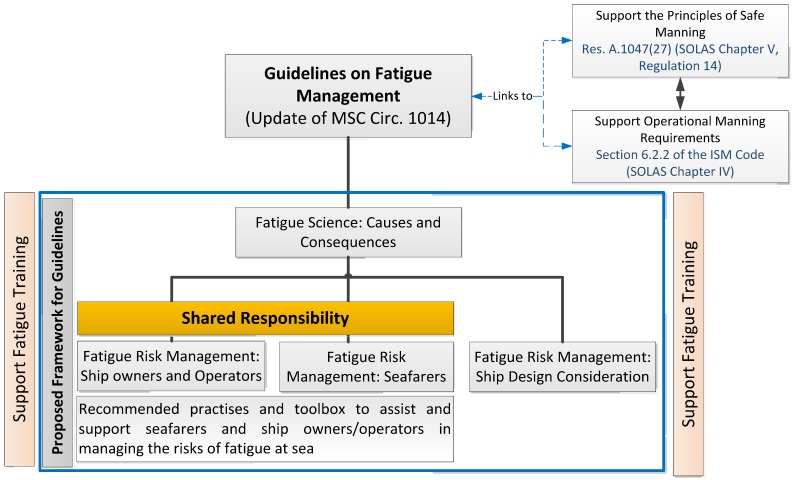
Proposed structure for IMO guidelines on fatigue [26].

Considering the view that a fatigue risk management approach in shipping for reducing the risks of fatigue at sea should be the way forward, Australia collaboratively with other interested parties to the IMO, initiated a process for reviewing and updating the current international Guidelines on Fatigue [25,26,27]. As a result of these initial submissions a proposed approach (Figure 1) and way forward for the new guidelines was presented [25] and discussed at the IMO. It was agreed that the need existed for a revision and update of the current IMO *Guidelines on fatigue mitigation and management* [27] and this needs to be carried out with a view that new guidelines consider a more holistic approach to managing the risks of fatigue at sea [25].

### 2.2. Proposed Maritime Fatigue Management Framework

As part of the review process a proposed maritime fatigue risk management framework was developed based on the principles agreed at the IMO, namely that:

- it should be risk based;
- it should consider ship design, company and operational aspects;
- it should consider the impact of fatigue at all levels; and
- the outcome should provide practical tools for fatigue management (suggested tools that can be easily applied in managing the risks of fatigue in practice).

**Figure 2:**
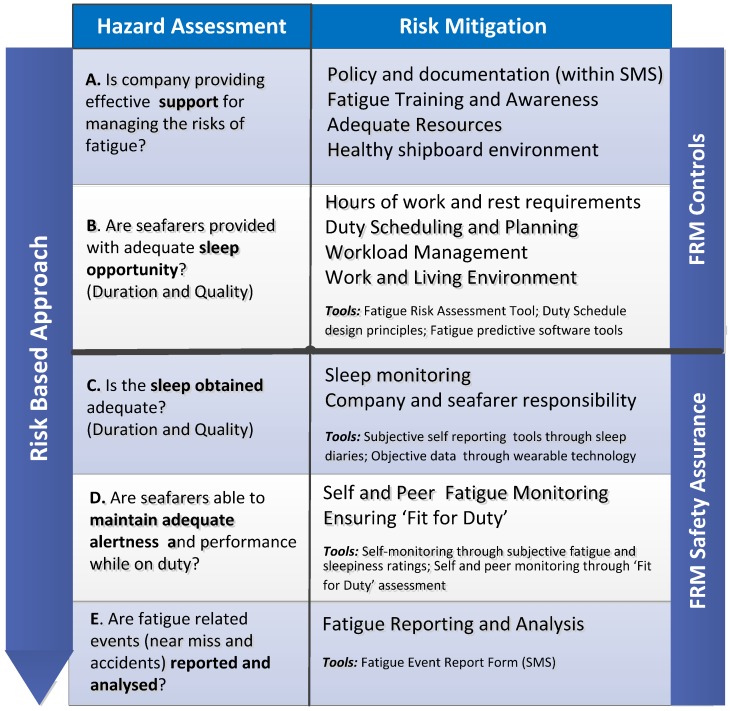
Maritime Fatigue Risk Management Framework [adapted from 18,23].

As shown in Figure 2, the proposed framework includes multiple layers of defences and associated control measures, primarily based on the “defences in depth” model [17,23,24,28]. This is composed of two important processes which are critical for its success and include fatigue risk management (FRM) controls and FRM safety assurance, within which are included appropriate layers of defences [29]. The FRM controls, effectively deal with the first two layers which are the principle mitigation strategies required to control and manage fatigue related risks:

- The first layer requires effective company support and commitment for managing and controlling the risks of fatigue;
- The second layer requires that seafarers are provided with adequate opportunity for sleep. This ensures that both duration and quality of sleep are considered.

The FRM safety assurance provides the data driven feedback (assessment and evaluation) through monitoring, to assure that the FRM controls are working effectively:

C. The third layer ensures that any issues affecting seafarers’ duration and quality of sleep, even though adequate opportunities for sleep have been provided, are being effectively captured. This entails monitoring and assessing sleep obtained and provides for the implementation of risk mitigation controls when issues are identified;
D. The fourth layer ensures that seafarers obtain what is considered, on average, sufficient sleep and are able to maintain adequate alertness and performance while performing their duties. This entails monitoring and assessing levels of fatigue and fitness for duty;
E. The fifth layer ensures that formal processes are in place for identifying and assessing fatigue related events or incidents. This layer relies on having an effective safety reporting culture (*i.e.*, just culture).

**Figure 3:**
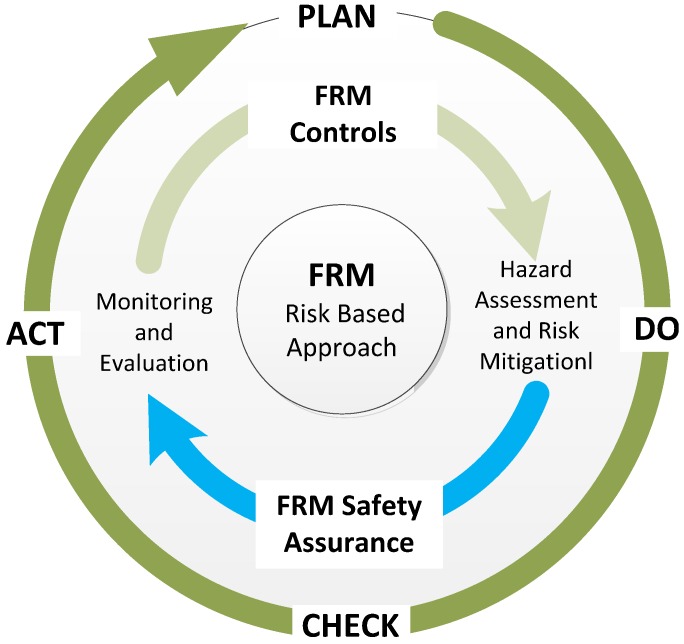
Maritime fatigue risk management: Continuous improvement process.

As shown in Figure 3, the combination of FRM controls and FRM safety assurance allows for continuous improvement within the maritime fatigue risk management framework [29]. This is not different to a Plan, Do, Check, Act approach. In general, if the controls perform to an acceptable standard (that is they bring the risk to as low as reasonably practicable) they become part of normal operations and are monitored and evaluated by the FRM safety assurance. If the controls do not perform to an acceptable standard, then it will be necessary to re-evaluate the controls at the appropriate step. As the company’s understanding of its own fatigue risk grows, through experience, it needs to be able to adjust and use the feedback driven by the safety assurance to improve the fatigue risk management processes to better manage fatigue.

### 2.3. Integrating within Shipboard Safety Management Systems

Fatigue risk management should not be standalone. As it has a safety function it should be integrated within existing shipboard Safety Management Systems (SMS). Part A, Sections 1.2.2.1 and 1.2.2.2 of the International Safety Management (ISM) Code states that the safety management objective should be to: (1) “Provide for safe practises in ship operation and a safe working environment”; and (2) “Assess all identified risks to its ships, personnel and the environment and establish appropriate safeguards” [30]. As fatigue is an operational safety risk then appropriate control measures should be implemented, managed, and assessed in accordance with the ship’s SMS objectives. Considering the current experience and level of maturity with shipboard safety management systems this allows fatigue to be managed within existing company safety structures, ensuring resources are appropriately distributed across the systems, maximising the effectiveness and efficiency of fatigue risk management.

## 3. Discussion

### Application in Practice

The first layer dealing with company support establishes the core risk control measure ensuring adequate level of support is available from the ship owner and/or operator for managing fatigue. The evidence is now clear from the *Shen Neng 1*, other accidents and supporting studies that this is lacking in the maritime domain [13,14]. Had the ship management company of the *Shen Neng 1* provided the necessary support including training, the crew would have been better informed about the causes and consequences of fatigue and possibly better prepared to implement more effective control measures.

The second layer ensures that current work scheduling practices are evaluated from a risk perspective. The company and management level officers on the *Shen Neng 1* would have had the opportunity to identify the issues associated with the high risk working patterns that the chief mate was engaged in and take appropriate action.

The third layer which is part of the FRM safety assurance processes provides the means, through data gathering and feedback, to identify, evaluate and implement new or revised controls and mitigation strategies. As highlighted in the *Shen Neng 1* and other fatigue related accidents the FRM safety assurance was a critical aspect missing in the company’s safety management system. Had the chief mate on the *Shen Neng 1* been encouraged to monitor his sleep, and through training would have been more knowledgeable about the issues regarding lack of sleep, he might have been in a better position to take appropriate action through feedback.

The fourth layer effectively deals with ensuring that seafarers who are not “fit for duty” are captured early with the ability that risk control measures can be put in place. On the *Shen Neng 1* this opportunity was available during the hand-over period through either peer monitoring or simply through conversation, and issues with fitness for duty may have been captured earlier.

The fifth layer of defence reactively identifies instances where fatigue related events have occurred and feeds this information back to strengthen higher level controls. It is possible that had the company and management level officers of the *Shen Neng 1* been encouraged in reporting and recording such events it would have kept them informed about fatigue hazards in day-to-day operations on the ship.

The aspect of “just culture” which flows through company support is crucial for this to work. The underlying culture in which fatigue risk management operates is a major component for successful implementation and management of fatigue-related risks. This is particularly important and in this context critical given the reliance on seafarers to report and provide feedback on not only fatigue related events but also their sleep and fatigue levels within and across days.

## 4. Summary and Conclusions

Recent years have seen a renewed interest in the area of fatigue management in shipping. This is also evident with the huge interest and traction this area is gaining from most stakeholders (such as companies, seafarer representatives, maritime administrations, classification societies). One of the challenges for effective fatigue risk management systems is the need for regulators and the maritime industry in general to have a sufficiently in depth knowledge and understanding of the causes and consequences of fatigue that enable them to meet their responsibilities in this regard [29]. As indicated, the industry needs more defensive layers than the hours of work and rest regulations to manage the risks of fatigue at sea.

Although the hours of work and rest regulation are here to stay, the implementation of a maritime fatigue risk management system as proposed in this paper will ensure effective management of the risks of seafarer fatigue with minimal impact on cost and operational flexibility. It is now widely accepted that fatigue can no longer be viewed as part of a labour or industrial relations [23] issues but as described, should be part of the ship’s safety management system and overall International Safety Management (ISM) Code. Recognising that there will be challenges in global wide acceptance and implementation, overall however, an effective fatigue risk management in shipping should bring the industry one step closer towards ensuring better health, well-being and safety for seafarers.
